# Validation List no. 216. Valid publication of new names and new combinations effectively published outside the IJSEM

**DOI:** 10.1099/ijsem.0.006229

**Published:** 2024-03-28

**Authors:** Aharon Oren, Markus Göker

**Affiliations:** 1 The Institute of Life Sciences, The Hebrew University of Jerusalem, The Edmond J. Safra Campus, 9190401 Jerusalem, Israel; 2 Leibniz Institute DSMZ – German Collection of Microorganisms and Cell Cultures, Inhoffenstrasse 7B, 38124 Braunschweig, Germany

The purpose of this list is to publish in the *International Journal of Systematic and Evolutionary Microbiology* (IJSEM) those new names and new combinations that were effectively published outside the IJSEM as set forth in Rule 27 of the International Code of Nomenclature of Prokaryotes (2022 Revision) [1]. Authors and other individuals wishing to have new names and/or combinations included in future lists should send an electronic copy of the published paper to the IJSEM Editorial Office for confirmation that all of the other requirements for valid publication have been met. It is also a requirement of IJSEM and the International Code of Nomenclature of Prokaryotes that authors of new species, new subspecies and new combinations provide evidence that types are deposited in two recognized culture collections in two different countries. It should be noted that the date of valid publication of these new names and combinations is the date of publication of this list, not the date of the original publication of the names and combinations. The authors of the new names and combinations are as given below. Inclusion of a name on these lists validates the publication of the name and thereby makes it available in the nomenclature of prokaryotes. Inclusion of a name on these lists is part of the requirements of valid publication of an effectively published name. Indeed, some of these names may, in time, be shown to be synonyms, or the organisms may be transferred to another genus, thus necessitating the creation of a new combination.

**Table:** 

Name/authors	Proposed as	Nomenclatural type^1^	Priority^2^	Reference
*Aceticella* Frolov *et al*. 2023, 17^3,4^	gen. nov.	*Aceticella autotrophica*	63	[2]
*Aceticella autotrophica* Frolov *et al*. 2023, 17^3^	sp. nov.	3443-3Ac (=DSM 108286=VKM B-3415)	63	[2]
*Actinoplanes aureus* Song *et al*. 2021, 1524	sp. nov.	NEAU-A11 (=CCTCC AA 2019063=JCM 33971)^5^	65	[3]
*Adlercreutzia aquisgranensis* Afrizal *et al*. 2022, e12^3^	sp. nov.	CLA-RA-2 (=DSM 108611=JCM 36500)	61	[4]
*Alsobacter ponti* Deng *et al*. 2023, 992	sp. nov.	SYSU M600028 (=CGMCC 1.19341=KCTC 92046)	60	[5]
*Ancylobacter crimeensis* Belova *et al*. 2023, 605^6^	sp. nov.	6 x-1 (=VKM B-3256=KCTC 92567)^7^	49	[6]
*Antrihabitans cavernicola* (Lee *et al*. 2020) Val-Calvo and Vázquez-Boland 2023, 15^3^	comb. nov. [basonym: *Rhodococcus cavernicola* Lee *et al*. 2020]	C1-24 (=DSM 109484=KACC 19964)	1	[7]
*Aquibacillus rhizosphaerae* Ding *et al*. 2023, 9^3^	sp. nov.	LR5S19 (=CGMCC 1.62028=KCTC 43434)	37	[8]
*Arthrobacter burdickii* Simpson *et al*. 2023, 9^3^	sp. nov.	IIF3SC-B10 (=DSM 115933=NRRL B-65660)	74	[9]
*Bacillus basilensis* Muigg *et al*. 2022, 7^3,8^	sp. nov.	403507–21 (=CCUG 75930=DSM 113537)	20	[10]
*Bacteroides rhinocerotis* Li *et al*. 2023, 7^3,9^	sp. nov.	NGMCC 1.200684 (=CGMCC 1.18013=JCM 35702)	16	[11]
*Billgrantia* de la Haba *et al*. 2023, 18^3^	gen. nov.	*Billgrantia desiderata*	54	[12]
*Billgrantia aerodenitrificans* de la Haba *et al*. 2023, 21^3^	sp. nov.	CYD-9 (=KCTC 72088=MCCC 1A11058)	54	[12]
*Billgrantia antri*(So *et al.* 2022) de la Haba *et al*. 2023, 21^3^	comb. nov. [basonym: *Halomonas antri* (So *et al*. 2022]	Y3S6 (=NBRC 114315=TBRC 15164)^10^	54	[12]
*Billgrantia azerbaijanica* (Kazemi *et al*. 2021) de la Haba *et al*. 2023, 20^3^	comb. nov. [basonym: *Halomonas azerbaijanica*Kazemi *et al*. 2021]	TBZ202 (=CECT 9693=KCTC 62817)	54	[12]
*Billgrantia bachuensis* (Xiao *et al*. 2021) de la Haba *et al*. 2023, 20^3^	comb. nov. [basonym: *Halomonas bachuensis*Xiao *et al*. 2021]	DX6 (=CCTCC AB 2020094=KCTC 82196)	54	[12]
*Billgrantia campisalis* (Mormile *et al*. 2000) de la Haba *et al*. 2023, 198	comb. nov. [basonym: *Halomonas campisalis*Mormile *et al*. 2000]	4A (=ATCC 700597=DSM 15413)^11^	54	[12]
*Billgrantia desiderata* (Berendes *et al*. 1997) de la Haba *et al*. 2023, 19^3^	comb. nov. [basonym: *Halomonas desiderata*Berendes *et al*. 1997]	FB2=DSM 9502=LMG 19548)^12^	54	[12]
*Billgrantia diversa* (Wang *et al*. 2021) de la Haba *et al*. 2023, 20^3^	comb. nov. [basonym: *Halomonas diversa*Wang *et al*. 2021]	D167-6-1 (=KCTC 72441=MCCC 1A13316)	54	[12]
*Billgrantia endophytica* (Chen *et al*. 2018) de la Haba *et al*. 2023, 19^3^	comb. nov. [basonym: *Halomonas endophytica*Chen *et al*. 2018]	MC28 (=KCTC 52999=MCCC 1K03343)	54	[12]
*Billgrantia ethanolica* de la Haba *et al*. 2023, 21^3^	sp. nov.	CYT3-1-1 (=KCTC 72090=MCCC 1A11081)	54	[12]
*Billgrantia gudaonensis* (Wang *et al*. 2007) de la Haba *et al*. 2023, 19^3^	comb. nov. [basonym: *Halomonas gudaonensis*Wang *et al*. 2007]	SL014B-69 (=DSM 23417=LMG 23610)^13^	54	[12]
*Billgrantia kenyensis* (Boltyanskaya *et al*. 2008) de la Haba *et al*. 2023, 19^3^	comb. nov. [basonym: *Halomonas kenyensis*Boltyanskaya *et al*. 2008]	AIR-2 (=DSM 17331=VKM B-2354)	54	[12]
*Billgrantia lactosivorans* (Ming *et al*. 2020) de la Haba *et al*. 2023, 20^3^	comb. nov. [basonym: *Halomonas lactosivorans*Ming *et al*. 2020]	CFH 90008 (=DSM 103220=KCTC 52281)	54	[12]
*Billgrantia montanilacus* (Lu *et al*. 2020) de la Haba *et al*. 2023, 20^3^	comb. nov. [basonym: *Halomonas montanilacus* Lu et al. 2020]	PYC7W (=CICC 24506=KCTC 62529)	54	[12]
*Billgrantia pellis* (Li *et al*. 2020) de la Haba *et al*. 2023, 20^3^	comb. nov. [basonym: *Halomonas pellis*Li *et al*. 2020]	L5 (=CGMCC 1.17335=KCTC 72573)	54	[12]
*Billgrantia saliphila* (Gan *et al*. 2018) de la Haba *et al*. 2023, 19^3^	comb. nov. [basonym: *Halomonas saliphila*Gan *et al*. 2018]	LCB169 (=CGMCC 1.15818=KCTC 52618)	54	[12]
*Billgrantia sulfidoxydans* de la Haba *et al*. 2023, 21^3^	sp. nov.	CYN-1–2 (=KCTC 72089=MCCC 1A11059)	54	[12]
*Billgrantia tianxiuensis* de la Haba *et al*. 2023, 22*	sp. nov.	BC-M4-5 (=KCTC 72092=MCCC 1A14433)	54	[12]
*Billgrantia zhangzhouensis* de la Haba *et al*. 2023, 22^3^	sp. nov.	CXT3-11 (=KCTC 72087=MCCC 1A11036)	54	[12]
*Bisbaumannia* de la Haba *et al*. 2023, 18^3^	gen. nov.	*Bisbaumannia pacifica*	54	[12]
*Bisbaumannia pacifica* (Baumann *et al*. 1972) de la Haba *et al*. 2023, 18^3^	comb. nov. [basonym: *Alcaligenes pacificus* corrig. Baumann *et al*. 1972]	62 (=DSM 4742=LMG 3446)^14^	54	[12]
*Cedecea sulfonylureivorans* Li *et al*. 2023, 5^3^	sp. nov.	LAM2020 (=GDMCC 1.2363=JCM 34640)	25	[13]
*Chryseobacterium herbae* Zhang *et al*. 2023, 10^3^	sp. nov.	pc1-10 (=GDMCC 1.3255=JCM 35711)	22	[14]
*Clostridium aromativorans* Luo *et al*. 2023, 745	sp. nov.	WKY-B-L2 (=CICC 25133=JCM 35127)	36	[15]
*Corynebacterium ramonii* Crestani *et al*. 2023, 7^3,15^	sp. nov.	FRC0011 (=CCUG 76912=CIP 112226)	52	[16]
*Desulfobotulus pelophilus* Frolova *et al*. 2023, 497^16^	sp. nov.	H1 (=DSM 112796=VKM B-3697)^17^	29	[17]
*Emticicia fluvialis* Baek *et al*. 2023, 1325	sp. nov.	BHSR1 (=GDMCC 1.3740=KCTC 92622)	50	[18]
*Enterobacter pseudoroggenkampii* Wu *et al*. 2023, 649	sp. nov.	155 092 (=GDMCC 1.3415=JCM 35646)	5	[19]
*Ferirhizobium* corrig. Romanenko *et al*. 2023, 13^3,18^	gen. nov.	*Ferirhizobium litorale* corrig.	32	[20]
*Ferirhizobium litorale* corrig. Romanenko *et al*. 2023, 13^3^	sp. nov.	KMM 9576 (=NRIC 0957)^19^	32	[20]
*Flintibacter muris* Afrizal *et al*. 2022, e16^3^	sp. nov.	CLA-AV-17 (=DSM 110149=JCM 36501)	61	[4]
*Franzmannia* de la Haba *et al*. 2023, 22^3^	gen. nov.	*Franzmannia pantelleriensis*	54	[12]
*Franzmannia pantelleriensis* (Romano *et al*. 1997) de la Haba *et al*. 2023, 22^3^	comb. nov. [basonym: *Halomonas pantelleriensis* Romano *et al*. 1997]	AAP (=DSM 9661=LMG 19550)^20^	54	[12]
*Franzmannia qiaohouensis* (Wang *et al*. 2015) de la Haba *et al*. 2023, 22^3^	comb. nov. [basonym: *Halomonas qiaohouensis*Wang *et al*. 2015]	YIM QH88 (=ACCC 60021=DSM 26770)^21^	54	[12]
*Fulvivirga sedimenti* Liu *et al*. 2022, 6^3^	sp. nov.	1062 (=KCTC 72868=MCCC 1H00499)	34	[21]
*Glycomyces luteolus* Duan *et al*. 2019, 707	sp. nov.	NEAU-A15 (=CGMCC 4.7394=DSM 104643)	66	[22]
*Glycomyces tritici* Li *et al*. 2018, 1091	sp. nov.	NEAU-C2 (=CGMCC 4.7410=DSM 104644)	67	[23]
*Gracilibacillus marinus* Huang *et al*. 2013, 699	sp. nov.	HB09003 (=CGMCC 1.10343=DSM 23372)	48	[24]
*Halorubrum hochsteinianum* Vreeland *et al*. 2024, 8^3^	sp. nov.	1–13–28 (=ATCC 700083=CGMCC 1.62627)	33	[25]
*Hymenobacter sediminicola* Ren *et al*. 2023, 823	sp. nov.	S2-20-2 (=CGMCC 1.18734=JCM 35801)	38	[26]
*Iodidimonas nitroreducens* Iino *et al*. 2023, 1^3^	sp. nov.	Q-1 (=JCM 17846=LMG 28992)	3	[27]
*Isoptericola croceus* OuYang *et al*. 2023, 850	sp. nov.	q2 (=GDMCC 1.2923=KCTC 49759)	59	[28]
*Lacinutrix neustonica* Choi *et al*. 2023, 10^3^	sp. nov.	HL-RS19 (=JCM 35710=KCCM 90497)	9	[29]
*Lactococcus intestinalis* Sun *et al*. 2023, 431^22^	sp. nov.	M2458 (=CGMCC 1.60066=JCM 35706)	21	[30]
*Leifsonia virtsii* Simpson *et al*. 2023, 11^3^	sp. nov.	F6_8S_P_1A (=DSM 115931=NRRL B-65661)	74	[9]
*Leifsonia williamsii* Simpson *et al*. 2023, 12^3^	sp. nov.	F6_8S_P_1B (=DSM 115932=NRRL B-65662)	74	[9]
*Litchfieldella* de la Haba *et al*. 2023, 23^3^	gen. nov.	*Litchfieldella anticariensis*	54	[12]
*Litchfieldella anticariensis* (Martínez-Cánovas *et al*. 2004) de la Haba *et al*. 2023, 23^3^	comb. nov. [basonym: *Halomonas anticariensis*Martínez-Cánovas *et al*. 2004]	FP35 (=CECT 5854=DSM 16096)^23^	54	[12]
*Litchfieldella qijiaojingensis* (Chen *et al*. 2012) de la Haba *et al*. 2023, 23^3^	comb. nov. [basonym: *Halomonas qijiaojingensis*Chen *et al*. 2012]	YIM 93003 (=DSM 22403=KCTC 22228)^24^	54	[12]
*Litchfieldella rifensis* (Amjres *et al*. 2011) de la Haba *et al*. 2023, 23^3^	comb. nov. [basonym: *Halomonas rifensis*Amjres *et al*. 2011]	HK31 (=CECT 7698=LMG 25695)	54	[12]
*Litchfieldella xinjiangensis* (Guan *et al*. 2010) de la Haba *et al*. 2023, 23^3^	comb. nov. [basonym: *Halomonas xinjiangensis*Guan *et al*. 2010]	TRM 0175 (=CCTCC AB 208329=KCTC 22608)	54	[12]
*Luteolibacter rhizosphaerae* Shen *et al*. 2023, 769	sp. nov.	GHJ8 (=GDMCC 1.2160=JCM 34400=KCTC 82452)	11	[31]
*Massilia cellulosilytica* Du *et al*. 2021, 1538	sp. nov.	NEAU-DD11 (=CCTCC AB 2019141=DSM 109721)	68	[32]
*Methanobacterium spitsbergense* Trubitsyn *et al*. 2023, 124^25^	sp. nov.	VT (=JCM 39284=VKM B-3566)	7	[33]
*Methanomethylophilus* Borrel *et al*. 2023, 9^3^	gen. nov.	*Methanomethylophilus alvi*	62	[34]
*Methanomethylophilus alvi* Borrel *et al*. 2023, 9^3^	sp. nov.	Mx-05 (=CIP 112449=JCM 31474)	62	[34]
*Methanomethylophilaceae* Borrel *et al*. 2023, 10^3^	fam. nov.	*Methanomethylophilus*	62	[34]
*Microbacterium helvum* Li *et al*. 2021, 3291	sp. nov.	NEAU-LLC (=CCTCC AA 2018026=JCM 32661)	69	[35]
*Micromonospora rubida* Sun *et al*. 2021, 705	sp. nov.	NEAU-HG-1 (=CGMCC 4.7479=JCM 32386)	70	[36]
*Modicisalibacter ilicicola* (Arenas *et al*. 2009) de la Haba *et al*. 2023, 24^3^	comb. nov. [basonym: *Halomonas ilicicola*Arenas *et al*. 2009]	SP8 (=CECT 7331=DSM 19980)^26^	54	[12]
*Modicisalibacter luteus* (Wang *et al*. 2008) de la Haba *et al*. 2023, 24^3^	comb. nov. [basonym: *Halomonas luteus*Wang *et al*. 2008]	YIM 91125 (=DSM 23508=KCTC 12847)^27^	54	[12]
*Modicisalibacter muralis* (Heyrman *et al*. 2002) de la Haba *et al*. 2023, 24^3^	comb. nov. [basonym: *Halomonas muralis*Heyrman *et al*. 2002]	R-5058 (=DSM 14789=LMG 20969)^28^	54	[12]
*Modicisalibacter radicis* (Navarro-Torre *et al*. 2020) de la Haba *et al*. 2023, 25^3^	comb. nov. [basonym: *Halomonas radicis* Navarro-Torre *et al*. 2020]	EAR18 (=CECT 9077=LMG 29859)	54	[12]
*Modicisalibacter xianhensis* (Zhao *et al*. 2012) de la Haba *et al*. 2023, 25^3^	comb. nov. [basonym: *Halomonas xianhensis*Zhao *et al*. 2012]	A-1 (=CGMCC 1.6848=JCM 14849)	54	[12]
*Modicisalibacter zincidurans* (Xu *et al*. 2013) de la Haba *et al*. 2023, 25^3^	comb. nov. [basonym: *Halomonas zincidurans*Xu *et al*. 2013]	B6 (=CGMCC 1.12450=JCM 18472)	54	[12]
*Muribaculum caecicola* Afrizal *et al*. 2022, e19^3^	sp. nov.	NM86_A22 (=DSM 110169=JCM 36504)	61	[4]
*Mycobacteriineae* Val-Calvo and Vázquez-Boland 2023, 15^3^	subord. nov.	*Mycobacterium*	1	[7]
*Mycoplasma bradburyae* Ramírez *et al*. 2023, 6^3^	sp. nov.	T158 (=DSM 110708=NCTC 14398)	18	[37]
*Natranaerovirgaceae* Sorokin and Merkel 2024, 1^3^	fam. nov.	*Natranaerovirga*	44	[38]
*Neiella litorisoli* Sun *et al*. 2023, 7^3^	sp. nov.	HB171785 (=KCTC 82319=MCCC 1K04625)	45	[39]
*Nocardiopsis akebiae* Mo *et al*. 2022, 5^3^	sp. nov.	HDS12 (=JCM 34708=MCCC 1K06173)	41	[40]
*Nocardiopsis changdeensis* Mo *et al*. 2023, 195	sp. nov.	Mg02 (=JCM 34709=MCCC 1K06174)^29^	42	[41]
*Nocardiopsis mangrovi* corrig. Huang *et al*. 2015, 1543^30^	sp. nov.	HA11166 (=CGMCC 4.7119=DSM 46665)	46	[42]
*Nonomuraea rhizosphaerae* Zhao *et al*. 2018, 2012	sp. nov.	NEAU-mq18 (=CGMCC 4.7431=DSM 105761)	71	[43]
*Nonomuraea sediminis* Liu *et al*. 2023, 8^3^	sp. nov.	H16431 (=CICC 25119=JCM 34852)	14	[44]
*Ochrobactrum soli* Choi *et al*. 2020, 1109	sp. nov.	BO-7 (=KACC 19676=LMG 30809)	58	[45]
*Odoribacter lunatus* Afrizal *et al*. 2022, e20^3^	sp. nov.	CLA-AA-M09 (=DSM 112344=JCM 36505)	61	[4]
*Onishia* de la Haba *et al*. 2023, 23^3^	gen. nov.	*Onishia taeanensis*	54	[12]
*Onishia niordana* (Diéguez *et al*. 2020) de la Haba *et al*. 2023, 24^3^	comb. nov. [basonym: *Halomonas niordana*Diéguez *et al*. 2020]	ATF 5.4 (=CECT 9779=LMG 31227)	54	[12]
*Onishia taeanensis* (Lee *et al*. 2005) de la Haba *et al*. 2023, 24^3^	comb. nov. [basonym: *Halomonas taeanensis*Lee *et al*. 2005]	BH539 (=DSM 16463=KCTC 12284)^31^	54	[12]
*Paenibacillus arenilitoris* Deng *et al*. 2022, 1314	sp. nov.	IB182493 (=JCM 34215=MCCC 1K04626)	47	[46]
*Paenibacillus aurantius* Hwang *et al*. 2023, 12^3^	sp. nov.	MBLB1776 (=JCM 34220=KCTC 43279)	64	[47]
*Paenibacillus vandeheii* Simpson *et al*. 2023, 12^3^	sp. nov.	F6_3S_P_1C (=DSM 115940=NRRL B-65663)	74	[9]
*Palleniella muris* Afrizal *et al*. 2022, e20^3^	sp. nov.	NM73_A23 (=DSM 110166=JCM 36503)	61	[4]
*Paracoccus onchidii* Xu *et al*. 2023, 812	sp. nov.	Z330 (=KCTC 92727=MCCC 1K08325)	28	[48]
*Parerythrobacter lacustris* Xamxidin *et al*. 2023, 4^3,32^	sp. nov.	RS5-5 (=GDMCC 1.3163=KCTC 92277)	19	[49]
*Pelosinus baikalensis* Zakharyuk *et al*. 2023, 143^33^	sp. nov.	Bkl1 (=JCM 39258=VKM B-3511)	15	[50]
*Planococcus lenghuensis* Yang *et al*. 2020, 846^34^	sp. nov.	Y42 (=CGMCC 1.15921=JCM 32719)	51	[51]
*Protaetiibacter mangrovi* Li *et al*. 2023, 537	sp. nov.	10F1B-8–1 (=CPCC 205428=JCM 33142)	6	[52]
*Pseudonocardia tritici* Song *et al*. 2019, 769	sp. nov.	NEAU-YY211 (=CGMCC 4.7474=DSM 106068)	72	[53]
*Pyrofollis* Miyazaki *et al*. 2023, 6^3^	gen. nov.	*Pyrofollis japonicus*	75	[54]
*Pyrofollis japonicus* Miyazaki *et al*. 2023, 6^3^	sp. nov.	YC29 (=DSM 113394=JCM 39171)	75	[54]
*Rhodalgimonas* corrig. Nedashkovskaya *et al*. 2023, 13^3,35^	gen. nov.	*Rhodalgimonas zhirmunskyi* corrig.	2	[55]
*Rhodalgimonas zhirmunskyi* corrig. Nedashkovskaya *et al*. 2023, 13^3,36^	sp. nov.	10Alg 79 (=KCTC 72611=KMM 6734)^37^	2	[55]
*Rhodococcoides* Val-Calvo and Vázquez-Boland 2023, 14^3^	gen. nov.	*Rhodococcoides fascians*	1	[7]
*Rhodococcoides corynebacterioides* (Serrano *et al*. 1972) Val-Calvo and Vázquez-Boland 2023, 14^3,38^	comb. nov. [basonym: *Nocardia corynebacterioides* Serrano *et al*. 1972 (Approved Lists 1980)]	DSM 20151 (=ATCC 14898)^39^	1	[7]
*Rhodococcoides fascians* (Tilford 1936) Val-Calvo and Vázquez-Boland 2023, 14^3,40^	comb. nov. [basonym: ‘*Phytomonas fascians*’ Tilford 1936]	DSM 20669 (=ATCC 12974)^41^	1	[7]
*Rhodococcoides kroppenstedtii* (Mayilraj *et al*. 2006) Val-Calvo and Vázquez-Boland 2023, 15^3^	comb. nov. [basonym: *Rhodococcus kroppenstedtii* Mayilraj *et al*. 2006]	K07-23 (=DSM 44908=JCM 13011)^42^	1	[7]
*Rhodococcoides kyotonense* (Li *et al*. 2007) Val-Calvo and Vázquez-Boland 2023, 15^3^	comb. nov. [basonym: *Rhodococcus kyotonensis* Li *et al*. 2007]	DS472 (=DSM 45159=JCM 23211)^43^	1	[7]
*Rhodococcoides trifolii* (Kämpfer *et al*. 2013) Val-Calvo and Vázquez-Boland 2023, 15^3^	comb. nov. [basonym: *Rhodococcus trifolii* Kämpfer *et al*. 2013]	T8 (=DSM 45580=LMG 26204)^44^	1	[7]
*Rhodococcoides yunnanense* (Zhang *et al*. 2005) Val-Calvo and Vázquez-Boland 2023, 15^3^	comb. nov. [basonym: *Rhodococcus yunnanensis* Zhang *et al*. 2005]	YIM 70056 (=JCM 13366=NBRC 103083)^45^	1	[7]
*Salinibacterium sedimenticola* Lu *et al*. 2023, 5^3^	sp. nov.	SYSU T00001 (=GDMCC 1.3283=KCTC 49758)	23	[56]
*Sedimenticola hydrogenitrophicus* Slobodkina *et al*. 2023, 9^3^	sp. nov.	SB48 (=KCTC 25568=VKM B-3680)	8	[57]
*Sedimenticolaceae* Slobodkina *et al*. 2023, 9^3^	fam. nov.	*Sedimenticola*	8	[57]
*Shewanella zhuhaiensis* Liu *et al*. 2023, 11^3^	sp. nov.	3B26 (=GDMCC 1.2057=KCTC 82339)	55	[58]
*Sphaerisporangium fuscum* Guo *et al*. 2022, 8^3^	sp. nov.	H8589 (=CICC 25115=JCM 34848)	13	[59]
*Sphingomonas nostoxanthinifaciens* Jiang *et al*. 2023, 10^3,46^	sp. nov.	AK-PDB1-5 (=CCTCC AB 2021150=KCTC 82822)	10	[60]
*Sporosarcina highlanderae* Simpson *et al*. 2023, 13^3^	sp. nov.	F6_3S_P_2 (=DSM 115943=NRRL B-65664)	74	[9]
*Staphylococcus brunensis* Kovařovic *et al*. 2023, 10^3,47^	sp. nov.	NRL/St 16/872 (=CCM 9024=DSM 111349=LMG 31872)	26	[61]
*Stieleria tagensis* Godinho *et al*. 2023, 1221	sp. nov.	TO1_6 (=CECT 30432=LMG 32465)	53	[62]
*Stratiformator* Kumar *et al*. 2023, 1004	gen. nov.	*Stratiformator vulcanicus*	4	[63]
*Stratiformator vulcanicus* Kumar *et al*. 2023, 1004	sp. nov.	Pan189 (=CECT 30699=DSM 101711)	4	[63]
*Streptococcus caecimuris* Afrizal *et al*. 2022, e23^3^	sp. nov.	CLA-AV-18 (=DSM 110150=JCM 36502)	61	[4]
*Streptomyces akebiae* Mo *et al*. 2022, 1302	sp. nov.	MG28 (=JCM 34922=MCCC 1K06895)	43	[64]
*Streptomyces anatolicus* Ates *et al*. 2023, 1085	sp. nov.	BG9H (=CGMCC 4.7699=DSM 110966)	27	[65]
*Streptomyces changanensis* Wu *et al*. 2024, 6^3,48^	sp. nov.	HL-66 (=CGMCC 22674=JCM 35800)	39	[66]
*Streptomyces spinosirectus* Wang *et al*. 2023, 4^3,49^	sp. nov.	CRSS-Y-16 (=JCM 35007=MCCC 1K06950)	12	[67]
*Streptomyces xiangluensis* Zhao *et al*. 2018, 2255	sp. nov.	NEAU-LA29 (=CGMCC 4.7466=DSM 105786)	73	[68]
*Sulfurospirillum tamanense* corrig. Frolova *et al*. 2023, 25^50^	sp. nov.	T05b (=DSM 112596=VKM B-3538)	30	[69]
*Thetidibacter* Yoon 2023, 638	gen. nov.	*Thetidibacter halocola*	17	[70]
*Thetidibacter halocola* Yoon 2023, 638	sp. nov.	KMU-90 (=KCCM 90287=NBRC 113375)	17	[70]
*Thiocapsa imhoffii* Asao *et al*. 2007, 674	sp. nov.	SC5 (=DSM 21303=NBRC 115608)^51^	40	[71]
*Thiomicrorhabdus marina* Tan *et al*. 2023, 8^3^	sp. nov.	6S2-11 (=KCTC 82994=MCCC 1H00523)	35	[72]
*Thiorhodovibrio frisius* Methner *et al*. 2023, 17^3^	sp. nov.	970 (=DSM 111777=KCTC 25638)	56	[73]
*Thiorhodovibrio litoralis* Methner *et al*. 2023, 16^3,52^	sp. nov.	06511 (=DSM 116345=KCTC 25737)	56	[73]
*Vreelandella* de la Haba *et al*. 2023, 11^3^	gen. nov.	*Vreelandella aquamarina*	54	[12]
*Vreelandella alkaliphila* (Romano *et al*. 2007) de la Haba *et al*. 2023, 14^3^	comb. nov. [basonym: *Halomonas alkaliphila*Romano *et al*. 2007]	18bAG (=ATCC BAA-953=DSM 16354)	54	[12]
*Vreelandella andesensis* (Guzmán *et al*. 2010) de la Haba *et al*. 2023, 15^3^	comb. nov. [basonym: *Halomonas andesensis*Guzmán *et al*. 2010]	LC6 (=CCUG 54844=DSM 19434)^53^	54	[12]
*Vreelandella aquamarina* (ZoBell and Upham 1944) de la Haba *et al*. 2023, 11^3^	comb. nov. [basonym: ‘*Achromobacter aquimarinus*’ ZoBell and Upham 1944]^54^	558 (=CECT 5000=DSM 30161)^55^	54	[12]
*Vreelandella arcis* (Xu *et al*. 2007) de la Haba *et al*. 2023, 13^3^	comb. nov. [basonym: *Halomonas arcis*Xu *et al*. 2007]	AJ282 (=DSM 23549=LMG 23978)^56^	54	[12]
*Vreelandella azerica* (Wenting *et al*. 2021) de la Haba *et al*. 2023, 17^3^	comb. nov. [basonym: *Halomonas azerica*Wenting *et al*. 2021]	TBZ9 (=KACC 21783=LMG 25775)	54	[12]
*Vreelandella boliviensis* (Quillaguamán *et al*. 2004) de la Haba *et al*. 2023, 13^3^	comb. nov. [basonym: *Halomonas boliviensis*Quillaguamán *et al*. 2004]	LC1 (=ATCC BAA-759=DSM 15516)	54	[12]
*Vreelandella glaciei* de la Haba *et al*. 2023, 17^3^	sp. nov.	DD 39 (=CGMCC 1.7263=JCM 11692)^57^	54	[12]
*Vreelandella gomseomensis* (Kim *et al*. 2007) de la Haba *et al*. 2023, 13^3^	comb. nov. [basonym: *Halomonas gomseomensis*Kim *et al*. 2007]	M12 (=DSM 18042=KCTC 12662)^58^	54	[12]
*Vreelandella hamiltonii* (Kim *et al*. 2010) de la Haba *et al*. 2023, 14^3^	comb. nov. [basonym: *Halomonas hamiltonii*Kim *et al*. 2010]	W1025 (=DSM 21196=KCTC 22154)	54	[12]
*Vreelandella janggokensis* (Kim *et al*. 2007) de la Haba *et al*. 2023, 13^3^	comb. nov. [basonym: *Halomonas janggokensis*Kim *et al*. 2007]	M24 (=DSM 18043=KCTC 12663)^59^	54	[12]
*Vreelandella jeotgali* (Kim *et al*. 2011) de la Haba *et al*. 2023, 15^3^	comb. nov. [basonym: *Halomonas jeotgali*Kim *et al*. 2011]	Hwa (=JCM 15645=KCTC 22487)	54	[12]
*Vreelandella lionensis* de la Haba *et al*. 2023, 18^3^	sp. nov.	RHS90 (=DSM 25632=UBOCC 3186)^60^	54	[12]
*Vreelandella lutescens* (Wang *et al*. 2016) de la Haba *et al*. 2023, 16^3^	comb. nov. [basonym: *Halomonas lutescens*Wang *et al*. 2016]	Q1U (=CGMCC 1.15122=KCTC 42517)	54	[12]
*Vreelandella malpeensis* (Kämpfer *et al*. 2018) de la Haba *et al*. 2023, 16^3^	comb. nov. [basonym: *Halomonas malpeensis*Kämpfer *et al*. 2018]	YU-PRIM-29 (=CCM 8737=LMG 28855)	54	[12]
*Vreelandella maris* de la Haba *et al*. 2023, 18^3^	sp. nov.	QX-1 (=KCTC 82198=NBRC 114670)^61^	54	[12]
*Vreelandella massiliensis* de la Haba *et al*. 2023, 18^3^	sp. nov.	Marseille-P2426 (=CSUR P2426=DSM 103116)	54	[12]
*Vreelandella nanhaiensis* (Long *et al*. 2013) de la Haba *et al*. 2023, 15^3^	comb. nov. [basonym: *Halomonas nanhaiensis*Long *et al*. 2013]	YIM M 13059 (=DSM 25561=JCM 18142)^62^	54	[12]
*Vreelandella neptunia* (Kaye *et al*. 2004) de la Haba *et al*. 2023, 12^3^	comb. nov. [basonym: *Halomonas neptunia*Kaye *et al*. 2004]	Eplume1 (=CECT 5815=DSM 15720)^63^	54	[12]
*Vreelandella nigrificans* (Oguntoyinbo *et al*. 2018) de la Haba *et al*. 2023, 16^3^	comb. nov. [basonym: *Halomonas nigrificans* Oguntoyinbo *et al*. 2018]	MBT G8648 (=DSM 105749=LMG 29097)	54	[12]
*Vreelandella olivaria* (Amouric *et al*. 2014) de la Haba *et al*. 2023, 15^3^	comb. nov. [basonym: *Halomonas olivaria*Amouric *et al*. 2014]	TYRC17 (=CCUG 53850 B=DSM 19074)	54	[12]
*Vreelandella piezotolerans* (Yan et al. 2020) de la Haba *et al*. 2023, 16^3^	comb. nov. [basonym: *Halomonas piezotolerans* Yan *et al*. 2020]	NBT06E8 (=KCTC 72680=MCCC 1K04228)	54	[12]
*Vreelandella populi* (Xu *et al*. 2022) de la Haba *et al*. 2023, 17^3^	comb. nov. [basonym: *Halomonas populi*Xu *et al*. 2022]	MC (=JCM 33545=MCCC 1K03942)	54	[12]
*Vreelandella profundi* (Wang *et al*. 2022) de la Haba *et al*. 2023, 17^3^	comb. nov. [basonym: *Halomonas profundi*Wang *et al*. 2022]	MT13 (=KCTC 82923=MCCC 1K06389)	54	[12]
*Vreelandella rituensis* (Gao *et al*. 2020) de la Haba *et al*. 2023, 16^3^	comb. nov. [basonym: *Halomonas rituensis*Gao *et al*. 2020]	TQ8S (=CICC 24572=KCTC 62530)	54	[12]
*Vreelandella salicampi* (Lee *et al*. 2015) de la Haba *et al*. 2023, 16^3^	comb. nov. [basonym: *Halomonas salicampi*Lee *et al*. 2015]	BH103 (=KACC 17609=NBRC 109914)^64^	54	[12]
*Vreelandella sedimenti* de la Haba *et al*. 2023, 18^3^	sp. nov.	QX-2 (=KCTC 82199=MCCC 1A17876)	54	[12]
*Vreelandella songnenensis* (Jiang *et al*. 2014) de la Haba *et al*. 2023, 16^3^	comb. nov. [basonym: *Halomonas songnenensis*Jiang *et al*. 2014]	NEAU-ST10-39 (=CGMCC 1.12152=DSM 25870)	54	[12]
*Vreelandella stevensii* (Kim *et al*. 2010) de la Haba *et al*. 2023, 14^3^	comb. nov. [basonym: *Halomonas stevensii*Kim *et al*. 2010]	S18214 (=DSM 21198=KCTC 22148)	54	[12]
*Vreelandella subglaciescola* (Franzmann *et al*. 1987) de la Haba *et al*. 2023, 12^3^	comb. nov. [basonym: *Halomonas subglaciescola*Franzmann *et al*. 1987]	ACAM 12 (=DSM 4683=JCM 21045)^65^	54	[12]
*Vreelandella subterranea* (Xu *et al*. 2007) de la Haba *et al*. 2023, 14^3^	comb. nov. [basonym: *Halomonas subterranea*Xu *et al*. 2007]	ZG16 (=DSM 23550=LMG 23977)^66^	54	[12]
*Vreelandella sulfidaeris* (Kaye *et al*. 2004) de la Haba *et al*. 2023, 13^3^	comb. nov. [basonym: *Halomonas sulfidaeris*Kaye *et al*. 2004]	Esulfide1 (=CECT 5817=DSM 15722)^67^	54	[12]
*Vreelandella titanicae* (Mann *et al*. 2010) de la Haba *et al*. 2023, 15^3^	comb. nov. [basonym: *Halomonas titanicae*Mann *et al*. 2010]	BH1 (=CECT 7585=DSM 22872)^68^	54	[12]
*Vreelandella utahensis* (Sorokin and Tindall 2006) de la Haba *et al*. 2023, 13^3^	comb. nov. [basonym: *Halomonas utahensis* Sorokin and Tindall 2006]	isolate III (=CECT 5286=DSM 3051)^69^	54	[12]
*Vreelandella venusta* (Baumann *et al*. 1972) de la Haba *et al*. 2023, 12^3^	comb. nov. [basonym: *Alkaligenes venustus* Baumann *et al*. 1972 (Approved Lists 1980)]	86 (=DSM 4743=LMG 3445)^70^	54	[12]
*Vreelandella vilamensis* (Menes *et al*. 2011) de la Haba *et al*. 2023, 15^3^	comb. nov. [basonym: *Halomonas vilamensis*Menes *et al*. 2011]	SV325 (=DSM 21020=LMG 24332)	54	[12]
*Vreelandella zhanjiangensis* (Chen *et al*. 2009) de la Haba *et al*. 2023, 14^3^	comb. nov. [basonym: *Halomonas zhanjiangensis*Chen *et al*. 2009]	JSM 078169 (=DSM 21076=KCTC 22279)^71^	54	[12]
*Vreelandella zhaodongensis* de la Haba *et al*. 2023, 17^3^	sp. nov.	NEAU-ST10-25 (=CGMCC 1.12286=DSM 25869)	54	[12]
*Vreelandella zhuhanensis* (Gao *et al*. 2020) de la Haba *et al*. 2023, 17^3^	comb. nov. [basonym: *Halomonas zhuhanensis*Gao *et al*. 2020]	ZH2S (=CICC 24505=KCTC 62531)	54	[12]
*Vulcanimicrobiaceae* Yabe *et al*. 2023, 1^3^	fam. nov.	*Vulcanimicrobium*	24	[74]
*Vulcanimicrobiales* Yabe *et al*. 2023, 1^3^	ord. nov.	*Vulcanimicrobium*	24	[74]
*Vulcanimicrobiia* Yabe *et al*. 2023, 1^3^	class. nov.^72^	*Vulcanimicrobium*	24	[74]
*Vulcanimicrobiota* Yabe *et al*. 2023, 1^3^	phyl. nov.	*Vulcanimicrobium*	24	[74]
*Weissella fermenti* Lee *et al*. 2023, 1454	sp. nov.	BK2 (=JCM 35750=KACC 22833)	57	[75]
*Weizmannia agrestimuris* Afrizal *et al*. 2022, e25^3^	sp. nov.	aMCA-6-a-A (=DSM 106041=JCM 36499)	61	[4]
*Winogradskyella immobilis* Wang *et al*. 2023, 6^3^	sp. nov.	E313 (=KCTC 82731=MCCC 1H00506)	31	[76]

For references to Validation Lists 1-71, see *Int J Syst Bacteriol* **49** (1999) 1325. Lists 72–197 were published in *Int J Syst Evol Microbiol* **50** (2000) 3, 423, 949, 1415, 1699, 1953; and **51** (2001) 1, 263, 793, 1229, 1619, 1945; and **52** (2002) 3, 685, 1075, 1437, 1915; and **53** (2003) 1, 373, 627, 935, 1219, 1701; and **54** (2004) 1, 307, 631, 1005, 1425, 1909; and **55** (2005) 1, 547, 983, 1395, 1743, 2235; and **56** (2006) 1, 499, 925, 1459, 2025, 2507; and **57** (2007) 1, 433, 893, 1371, 1933, 2449; and **58** (2008) 1, 529, 1057, 1511, 1993, 2471; and **59** (2009) 1, 451, 923, 1555, 2129, 2647; and **60** (2010) 1, 469, 1009, 1477, 1985, 2509; **61** (2011) 1, 475,1011, 1499, 2025, 2563; and **62** (2012) 1, 473, 1017, 1443, 2045, 2549; and **63** (2013) 1, 797, 1577, 2365, 3131, 3931; and **64** (2014) 1, 693, 1455, 2184, 3603; and **65** (2015), 1, 741, 1112, 2017, 2777, 3767; and **66** (2016) 1, 1603, 1913, 2463, 3761, 4299; and **67** (2017) 1, 529, 1095, 2075, 3140, 4291; and **68** (2018), 1, 693, 1411, 2130, 2707, 3379; and **69** (2019), 5, 597, 1247, 1844, 2627, 3313 and **70** (2020) 1, 1443, 2960, 4043, 4844, 5596. Lists 197–215 were published in *Int J Syst Evol Microbiol* **71** (2021) 004600, 004688, 004773, 004846, 004943, 005096; and **72** (2022) 005167, 005260, 005331, 005422, 005517, 005592; and **73** (2023) 005709, 005812, 005845, 005931, 005997, 006080; and **74** (2024) 006173.

1Abbreviations of culture collections cited in this list can be found at https://lpsn.dsmz.de/text/collections

2Priority number assigned according to the date the documentation and request for validation are received.

3The journal in which the name was effective published does not have continuous pages.

4The preferred etymology is: L. neut. n. *acetum*, vinegar; L. fem. n. *cella*, cell; N.L. fem. n. *Aceticella*, vinegar cell.

5The CCTCC number is erroneously given as AA 2,019,063 in the protologue.

6The etymology should state N.L. masc. adj. instead of N.L., adj.

7The effective publication states that the type strain was also deposited as CCUG 72401, but no documentation was supplied.

8The etymology should state N.L. masc. adj. instead of N.L. masc./fem. adj. The protologue is found above the etymology line.

9The preferred syllabification is rhi.no.ce.ro’tis.

10The effective publication states that the type strain was also deposited as KACC 21536, but no documentation was supplied.

11The effective publication states that the type strain was also deposited as CIP 106639, but no documentation was supplied.

12The effective publication states that the type strain was also deposited as CIP 105505, but no documentation was supplied.

13The effective publication states that the type strain was also deposited as CGMCC 1.6133, but no documentation was supplied.

14The effective publication states that the type strain was also deposited as ATCC 27122, CIP 103200, JCM 20633, NBRC 102220 and NCIMB 1977, but no documentation was supplied.

15The protologue heading should show the full spelling *Corynebacterium ramonii*; the preferred etymology is ra.mo’ni.i. N.L. gen. n. *ramonii*, named in honour of Gason Ramon,…

16The preferred etymology is Gr. masc. n. *pelos*, mud; N.L. masc. adj. *philus* (from Gr. masc. adj. *philos*), loving; N.L. masc. adj. *pelophilus*, mud-loving.

17The effective publication states that the type strain was also deposited as UQM 41590, but no documentation was supplied.

18The list editors have corrected *Fererhizobium* to *Ferirhizobium* (Fe.ri.rhi.zo’bi.um. L. adj. *fere*, nearly, almost; N.L. neut. n. *Rhizobium*, a bacterial genus name; N.L. neut. n. *Ferirhizobium*, a genus adjacent to *Rhizobium*).

19The effective publication states that the type strain was also deposited as NRIC 0957, but no documentation was supplied.

20The effective publication states that the type strain was also deposited as ATCC 700273 and CIP 105506, but no documentation was supplied.

21The effective publication states that the type strain was also deposited as CCTCC AB 2012965, but no documentation was supplied.

22The preferred syllabification and etymology is in.tes.ti.na’lis. N.L. masc. adj. *intestinalis*, intestinal.

23The effective publication states that the type strain was also deposited as CIP 108499 and LMG 22089, but no documentation was supplied.

24The effective publication states that the type strain was also deposited as CCTCC AB 208133, but no documentation was supplied.

25The preferred syllabification and etymology is spits.ber.gen’se. N.L. neut. adj. *spitsbergense*, pertaining to Spitsbergen.

26The effective publication states that the type strain was also deposited as CCM 7522, but no documentation was supplied.

27The effective publication states that the type strain was also deposited as CCTCC AB 206093, but no documentation was supplied.

28The effective publication states that the type strain was also deposited as CIP 108825, but no documentation was supplied.

29The culture collection numbers given in the lines above the protologue and in the abstract.

30The list editors have corrected *mangrovei* to *mangrovi* (man.gro’vi. N.L. gen. n. *mangrovi*, of a mangrove).

31The effective publication states that the type strain was also deposited as CIP 109003, but no documentation was supplied.

32The etymology should state N.L. masc. adj. instead of N.L. masc./fem. adj.

33The etymology should state N.L. masc. adj. instead of N.L. neut. adj.

34The protologue heading should give *Planococcus lenghuensis* sp. nov. instead of *planococcus lenghuensis* sp. Nov.

35The list editors have corrected *Rhodoalgimonas* to *Rhodalgimonas* (Rhod.al.gi.mo’nas. Gr. neut. n. *rhodon*, rose; L. fem. n. *alga*, alga, seaweed; L. fem. n. *monas*, a monad, unit; N.L. fem. n. *Rhodalgimonas*, a red (rose) monad isolated from seaweed).

36The etymology should be corrected to: zhir.mun’sky.i. N.L. gen. n. *zhirmunskyi*, named in honour of the academician A.V. Zhirmunsky…

37The designated type and the culture collection accession numbers were mentioned in the abstract but not in the protologue.

38The etymology should state N.L. neut. adj.

39The effective publication states that the type strain was also deposited as CCUG 37877, CIP 104510, IFO 14404, JCM 3376, JCM 3391, NBRC 14404 and NRRL B-24037, but no documentation was supplied.

40The preferred syllabification is fas’ci.ans.

41The effective publication states that the type strain was also deposited as CFBP 2401, CIP 104713, ICMP 5833, IFO 12155, JCM 10002, LMG 3623, NBRC 12155, NCPPB 3067, NRRL B-16937 and VKM Ac-1462, but no documentation was supplied.

42The effective publication states that the type strain was also deposited as MTCC 6634 and NBRC 103113, but no documentation was supplied.

43The effective publication states that the type strain was also deposited as CCTCC AB 206088 and IAM 15415, but no documentation was supplied.

44The effective publication states that the type strain was also deposited as CCM 7905, but no documentation was supplied.

45The effective publication states that the type strain was also deposited as CCCTCC AA 204007, DSM 44837, KTC 19021 and NBRC 103115, but no documentation was supplied.

46The etymology should state N.L. part. adj. instead of N.L. part. Adj.

47The protologue heading should state *Staphylococcus brunensis* instead of *S. brunensis*. The etymology should state L. masc. adj. instead of L. adj.

48The etymology should state N.L. masc. adj. instead of N.L. adj.

49The epithet was also given as *Spinosirectus* in the article title and protologue heading, contravening Rule 59.

50The list editors have corrected the epithet to *tamanense* (ta.ma.nen’se. N.L. neut. adj. *tamanense*, living in Taman).

51The effective publication states that the type strain was also deposited as ATCC BAA-1499, but no documentation was supplied.

52The etymology should state L. masc. adj. instead of L. adj.

53The effective publication states that the type strain was also deposited as LMG 24243, but no documentation was supplied.

54The nomenclatural authority is ZoBell and Upham 1944 and not ZoBeAll and Upham 1944 as given by the authors.

55The effective publication states that the type strain was also deposited as ATCC 14400, BCRC 12878, CCUG 16157, CGMCC 1.2324, CIP 105454, IAM 12550, KCTC 22193, LMD 73.17, LMG 2853 and NCIMB 557, but no documentation was supplied.

56The effective publication states that the type strain was also deposited as CGMCC 1.6494 and JCM 14607, but no documentation was supplied.

57The effective publication states that the type strain was also deposited as MTCC 4321, but no documentation was supplied.

58The effective publication states that the type strain was also deposited as CIP 109897, but no documentation was supplied.

59The effective publication states that the type strain was also deposited as CIP 109896, but no documentation was supplied.

60The effective publication states that the type strain was also deposited as CIP 110370, but no documentation was supplied.

61The effective publication states that the type strain was also deposited as MCCC 1A17875, but no documentation was supplied.

62The effective publication states that the type strain was also deposited as CCTCC AB 2012911, but no documentation was supplied.

63The effective publication states that the type strain was also deposited as ATCC BAA-805 and CCM 7107, but no documentation was supplied.

64The effective publication states that the type strain was also deposited as NCAIM B 02528, but no documentation was supplied.

65The effective publication states that the type strain was also deposited as ATCC 43668, CIP 104042, IAM 14167, LMG 8824, NBRC 14766 and UQM 2926, but no documentation was supplied.

66The effective publication states that the type strain was also deposited as CIP 109673, CGMCC 1.6495 and JCM 14608, but no documentation was supplied.

67The effective publication states that the type strain was also deposited as ATCC BAA-803 and CCM 7108, but no documentation was supplied.

68The effective publication states that the type strain was also deposited as ATCC BAA-1257, JCM 16411 and LMG 25388, but no documentation was supplied.

69The effective publication states that the type strain was also deposited as ATCC 49240, CIP 105504, AM 14440, JCM 21223 and NBRC 102410, but no documentation was supplied.

70The effective publication states that the type strain was also deposited as ATCC 27125, CCUG 16063, CIP 103201, JCM 20634 and NBRC 102221, but no documentation was supplied.

71The effective publication states that the type strain was also deposited as CCTCC AB 208031, but no documentation was supplied.

72The list editors corrected *Vulcanimicrobiales* to *Vulcanimicrobium* as the nomenclatural type of a class is one of the contained genera, in accordance with the emendations of Rules 15 and 22 that took effect on 11 September 2023 [77].
